# Characterization of patients with massive hypertrophic cardiomyopathy using contrast-enhanced magnetic resonance imaging: does contrast provide additional information?

**DOI:** 10.1186/1532-429X-14-S1-O99

**Published:** 2012-02-01

**Authors:** Raymond H Chan, Susie Hong, Tammy S Haas, Kristin Feeney, John Lesser, Michael C Gibson, Warren J Manning, Barry J Maron, Martin Maron, Evan Appelbaum

**Affiliations:** 1 cardiology, BIDMC, Boston, MA, USA; 2 Tufts Medical Center, Boston, MA, USA; 3 Minneapolis Heart Institute Foundation, Minneapolis, MN, USA

## Background

Hypertrophic cardiomyopathy (HCM) patients with massive left ventricular hypertrophy (LVH) (hereby defined as maximal LV wall thickness >30mm) are known to have lower rates of survival. Recently, contrast-enhanced cardiac magnetic resonance (CMR) with late gadolinium enhancement (LGE) has emerged as a potential novel marker of increased cardiovascular risk in HCM.

## Methods

Cine CMR and LGE imaging were performed on HCM patients with massive LV hypertrophy and compared with those with a maximal LV wall thickness <30mm.

## Results

Among 902 consecutive HCM patients, 40 (4.4%) had a maximal LV wall thickness of ≥ 30mm (mean thickness 33.7± 4.2mm, range 30 - 50mm, 23% females) with a mean age of 37 ± 18 years old, including 11 patients (28%) ≥ 50 years of age. When compared to those with a maximal wall thickness less than 30mm, these patients were significantly younger (mean age 37 ± 18 vs 47 ± 18, p<0.001) and had a similar proportion of males (77.5% versus 66.8%, p=0.16). Mean left ventricular mass was significantly higher (317 ± 142 versus 163 ± 62 grams, p<0.001). They also had significantly higher LV end diastolic volume (LVEDV) (169.6 ± 58.5 versus 153.0 ± 42.1 mL, p<0.01) and LV end systolic volume (LVESV) (63.0± 40.8 versus 49.9 ± 23.1 ml, p=0.001). They had similar LV ejection fraction (64.8 ± 10.7% versus 67.6 ± 9.6%, p=0.21), LVEDD (51.7 ± 9.2mm versus 53.1 ± 6.8mm, p=0.21), and stroke volume (106.6 ± 27.5 ml versus 103.1 ± 30.8 ml, p=0.48).

LGE was identified in 95% of HCM patients with massive LVH, occupying an average of 19.6 g ±19.8 g (or 6.1 ± 4.7%) of LV myocardial mass. 18% had LGE occupying>10% of the LV. In comparison, only 38% of HCM patients without massive LVH had LGE (p<0.001), occupying an average of only 13.6 ± 17.0 g (or 7.8 ± 9.6%) of LV myocardial mass (p=0.05).

Univariate associates of massive hypertrophy include young age (OR=1.37/decade decrease in age, p<0.001 ), presence of LGE (OR 25.6, p<0.001), amount of LGE present (OR = 1.31 per 10g increase, p<0.001), presence of LV dysfunction (defined as LVEF<50%) (OR 3.28, p=0.01) , LVEDV (OR =1.08 per 10 ml increase in LVEDV, p=0.018), and LVESV (OR = 1.16 per 10 ml increase in LVESV, p=0.001). On multivariate regression analysis, presence of LGE (adjusted OR 21.3, p<0.0001) and young age (adjusted OR =1.43/decade decrease in age, p=0.0014) remained significant after controlling for other factors.

When comparing patients older and young than 30 years old with massive hypertrophy, there were no significant differences in LV mass, stroke volume or quantity of LGE (p>0.10). Older patients had a smaller cavity size (p=0.04) and better cardiac function ( LVEF =67.7 ± 9.8% versus 60.8 ± 10.9%, p=0.046).

## Conclusions

Almost all HCM patients with massive LV hypertrophy demonstrate LGE. These observations may complement findings demonstrating an increased risk with LGE. Patients with massive LVH surviving to older age had better cardiac function and less dilation.

## Funding

n/a.

